# DCP-SLAM: Distributed Collaborative Partial Swarm SLAM for Efficient Navigation of Autonomous Robots

**DOI:** 10.3390/s23021025

**Published:** 2023-01-16

**Authors:** Huma Mahboob, Jawad N. Yasin, Suvi Jokinen, Mohammad-Hashem Haghbayan, Juha Plosila, Muhammad Mehboob Yasin

**Affiliations:** 1 Autonomous Systems Laboratory, Department of Future Technologies, University of Turku, Vesilinnantie 5, 20500 Turku, Finland; 2 ABB Oy, 00380 Helsinki, Finland; 3 Department of Computer Networks, College of Computer Sciences & Information Technology, King Faisal University, Hofuf 31982, Saudi Arabia

**Keywords:** swarm robotics, collaborative sensing, multi-agent systems, energy efficient, swarm intelligence, leader–follower, collision avoidance

## Abstract

Collaborative robots represent an evolution in the field of swarm robotics that is pervasive in modern industrial undertakings from manufacturing to exploration. Though there has been much work on path planning for autonomous robots employing floor plans, energy-efficient navigation of autonomous robots in unknown environments is gaining traction. This work presents a novel methodology of low-overhead collaborative sensing, run-time mapping and localization, and navigation for robot swarms. The aim is to optimize energy consumption for the swarm as a whole rather than individual robots. An energy- and information-aware management algorithm is proposed to optimize the time and energy required for a swarm of autonomous robots to move from a launch area to the predefined destination. This is achieved by modifying the classical Partial Swarm SLAM technique, whereby sections of objects discovered by different members of the swarm are stitched together and broadcast to members of the swarm. Thus, a follower can find the shortest path to the destination while avoiding even far away obstacles in an efficient manner. The proposed algorithm reduces the energy consumption of the swarm as a whole due to the fact that the leading robots sense and discover respective optimal paths and share their discoveries with the followers. The simulation results show that the robots effectively re-optimized the previous solution while sharing necessary information within the swarm. Furthermore, the efficiency of the proposed scheme is shown via comparative results, i.e., reducing traveling distance by 13% for individual robots and up to 11% for the swarm as a whole in the performed experiments.

## 1. Introduction

Swarm robotics is a field inspired by natural self-organizing swarms, such as birds, bees, ants, and fish [1]. The aim of researchers is to create swarms of autonomous robots that can mimic such self-organizing behavior in different situations to carry out their collective mission in an energy-efficient manner [2]. Each robot in the swarm executes relatively simple control routines to accomplish its task. It uses its onboard sensors for awareness of the surrounding environment, whereas it relies on wireless messaging for coordination with other robots of its formation, akin to behavior of individual agents in a multi-agent system [3]. In the following text, the terms agent and robot are used interchangeably. Due to their small size, robustness, and ability to reach difficult or hazardous environments, there has been exponential increase in research for the development of novel techniques for the integration of autonomous robots in various applications, such as surveillance [4], infrastructure inspection [5], military applications [6], GPS-denied environments [7], transportation [8], hazardous environments [9], and mapping or atmospheric research [10].

Of particular interest is the problem of navigation of a swarm of autonomous robots in an unknown environment, such as a GPS-denied area or where no prior map information exists. Work in this field presents a wide array of research challenges, such as the ability to maintain formation, self-localization, collision avoidance, and path finding [3]. Simultaneous localization and mapping (SLAM) is a classical and fundamental technique for mapping and localizing in the field of autonomous robots in environments with no prior map information. Until relatively recently, SLAM development was mainly concerned with a single agent or robot, hence leaving a significant gap for the development of SLAM techniques with multiple agents or a swarm of robots [11,12]. Various collaborative SLAM (C-SLAM) methodologies and techniques have been researched, where the primary objective has been to integrate the data gathered by individual robots to build a global map that is shared between all robots [13]. In collaborative navigation, the actions of one agent may impact those of other agents in the system; therefore, it is vital to realize how the collaborative navigation can be of assistance. The coordination strategies to attain autonomy for a swarm of agents can be categorized into the following three types [14,15]: (1) centralized coordination, in which either a central node or server or an established agent, often labeled as a dedicated leader, provides the vital parameters to the rest of the agents in the swarm, such as maneuver information, trajectory planning, and coordination; these are often described as virtual structure-based approaches or leader–follower-based approaches [16]; (2) decentralized with coordination-based approaches, in which all agents in the swarm can directly interact with other agents within their communication range; (3) decentralized without coordination-based approaches, in which the agents do not interact with each other to interchange the individually gathered data; in this approach, the agents work on the observe and react principle [17].

The sensing modes utilized for C-SLAM can be divided into two broad categories, namely, vision-based and laser/LiDAR-based. Due to their high resolution, robustness to varying weather conditions, and resilience against lighting conditions, several LiDAR C-SLAM techniques have been investigated [18,19,20,21,22]. Approaches presented in [18,20] work by transmitting the locally generated maps of all the robots to a central base station/server for optimization and stitching purposes. This process requires high availability of the central server, the failure of which results in the failure of the entire mission. Furthermore, this approach is very dependent on the communication channel between each robot and the server, requiring it to be lossless and have high bandwidth. To alleviate the dependence on a lossless communication channel, ref. [21] presented a three-dimensional LiDAR-based data-driven descriptor approach to optimize the required transmission bandwidth. The presented methodology functions on a completely centralized system; it is not suitable for larger swarms. Most of the present C-SLAM-based approaches are either highly centralized or the database is split into several segments for assessment,  have high computational costs, or require higher communication bandwidth.

**Figure 1:**
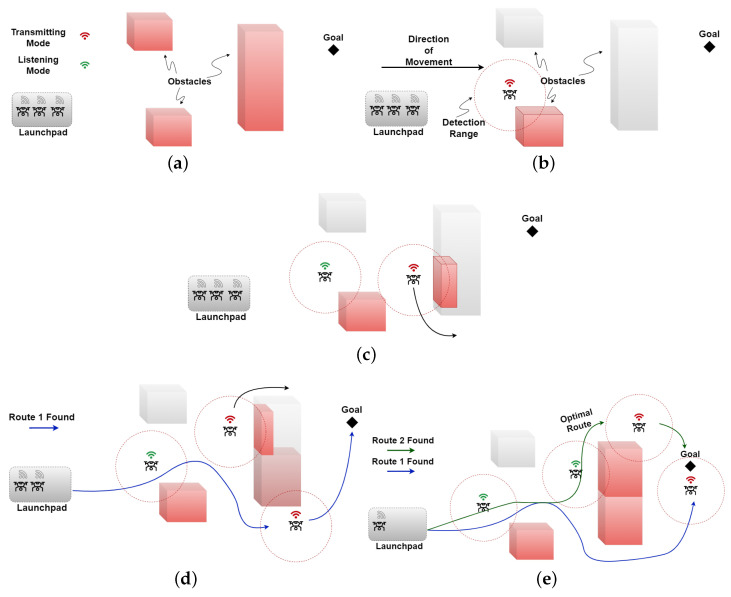
Illustration of DCP-SLAM technique. Unknown obstacles are illustrated in grey. Once an obstacle (or a part of it) is detected by the onboard sensors of the robot(s), it is illustrated in red. (**a**) Initial setup, launchpad where robots are launched from and unknown obstacles are shown. (**b**) Robot 1 detects an obstacle while navigating towards goal. Robot 1 broadcasts the information to following robots. (**c**) Partial detection of second obstacle by Robot 1, Robot 2 navigating towards the waypoint dropped by Robot 1. (**d**) Robot 1 finds unobstructed path to goal, Robot 2 navigates from the other side of the obstacle, while broadcasting the information. (**e**) Robot 2 finds unobstructed path to goal. Followers opt for the optimal path.

Minimization of the energy consumption of a swarm as a whole is another important research area with core emphasis on a varied set of themes, such as dealing with external influences [23,24], optimization of consumption due to ranging sensors [25], efficient inter-robot communication [26], optimization of distance to be traveled [27,28], or recharging optimization [29,30]. In this respect, we present DCP-SLAM, a LiDAR-based distributed collaborative partial SLAM framework for swarm robotics. In the proposed DCP-SLAM technique, we focus on energy efficiency of the swarm as a whole rather than trying to accurately map individual obstacles. We argue that the requirement of maintaining a safe distance from obstacles allows us to conduct trajectory planning even with partially discovered obstacles. Moreover, a cluster of obstacles where interobstacle distance does not allow a robot to pass between them can be fuzzified into one large obstacle without causing excessive elongation of the collision-free path. Furthermore, this fuzzification reduces communication cost as less data need to be transmitted. Thus, in order to have a low-bandwidth low-computation navigation setup for the swarm, an exact replication of the map is not required to be communicated between the robots. Furthermore, the safe distance requirement, also referred to as inflation, since each obstacle’s dimensions are inflated by this amount, covers up the sacrificed error while performing active obstacle detection and avoidance maneuvers. This is handled in three phases: (i) The first is the detection and map building phase, in which the point cloud returned by LiDAR after each scan is utilized to update the obstacle list. Here, we adapt the Euclidean-distance-based incremental region growing technique of [31] by adding fuzzification while calculating distances. The concept of fuzzification and merging of objects is explained in Figure 1. The resulting obstacle list is broadcast to the rest of the swarm. (ii) In the second phase, the robot generates waypoints for bypassing all known obstacles in the updated obstacle list. (iii) Finally, the robot will align itself in order to move towards the nearest waypoint as a temporary goal. Since the obstacle list only contains information about obstacles that have been fully or partially discovered so far, hitherto unknown obstacles may still be present and in the way of a robot. Therefore, the robots are required to continually perform scanning and obstacle detection during navigation. Simulations of various scenarios show that the DCP-SLAM technique results in swarms where later robots show marked improvement in their trajectory by utilizing partial maps discovered by their earlier fellows. Even in complex scenarios, the optimal path is established relatively quickly.

The organization of the rest of the paper is as follows: Section 2 provides the related work and motivation for DCP-SLAM. Section 3 explains working of the proposed algorithm in detail. Simulation results are provided in Section 4. Finally, concluding remarks, discussion, and future work is presented in Section 5.

## 2. Related Work

Navigation in environments with no prior map information raises several research challenges. The autonomous robots or agents, while navigating in unknown environments and not having to bank on acquiring information from central or remote servers, must utilize their onboard sensors to observe, analyze, and perform necessary actions based on the information at hand for collision-free navigation and successful completion of the mission [25]. At the same time, reducing the power consumed by the autonomous agents, i.e., effectively exploiting the available resources, in order to increase the mission duration is of paramount importance [32]. With reference to the existing literature, the proposed approach relates closely to the detection, avoidance, and energy-efficient path development for the swarm as a whole.

In SLAM, exploration is fundamental, and its importance becomes even more vital in swarm SLAM systems (or multi-robot SLAM). Various exploration methodologies are utilized to facilitate the exploration, such as random walk exploration [33,34], potential field-based or frontier-based exploration; however, path planning is the most commonly utilized scheme [35]. Authors in [34] utilized the random walk exploration scheme for a robot swarm mapping event, and illustrated that an occupancy grid can be produced in a closed indoor environment. However, in practical situations, the proposed methodology struggles to work efficiently due to the poor quality of the close-range sensors. Furthermore, by employing high-precision sensors, this issue can be rectified to virtually produce any kind of map, as shown in the experiment performed by the authors in [36]. Ideally, the robots in the swarm should be as simple as possible, and should employ simple algorithms with low overhead, due to practicality. Therefore, swarm SLAM schemes that are able to make use of low-cost and low-precision sensors and generating relatively abstract maps for navigational purposes are of greater importance. Authors in [37] demonstrated such an approach for generating semantic maps; however, the computational complexity of the proposed approach is significantly high for real-world scenarios for swarms. Such an issue, with focus on map retrieval without centralization, is an open problem in the field of swarm SLAM. As shown in [34], a natural technique for merging the maps to be utilized by individual robots of the swarm is to accumulate the individual maps on a single central system. Furthermore, another approach proposed by [11] works by merging individually generated maps in all robots in the swarm. In this manner, every robot or agent in the swarm has access to the map; however, for the successful deployment of such an approach, an external framework or central node is necessary.

Literature differentiates collaborative SLAM (C-SLAM) as centralized or decentralized architectures, with centralized schemes gathering all the data into a central server (central station or a robot) and assessing the trajectories for all the robots. Different sensing arrangements, such as laser or LiDAR-based [18,38] and vision-based [39,40], are examined in the centralized C-SLAM. In [38], locally generated maps constructed via utilizing LiDARs are sliced, and all the segments of the sliced data are accumulated for the detection of loop closure. The authors of [18] introduced a large-scale autonomous mapping and positioning system (LAMP) utilizing a 3D LiDAR scanner mounted on single or multiple robots to scan the surroundings, and an RGB-D camera for the detection and localization of known obstacles in the environment. In the proposed scheme, all the robots in the swarm are connected to a central server or node, and in case of loss of communication with the base station, the algorithm switches back to classical single-robot SLAM-based navigation. For vision-based techniques, e.g., the works presented in [39,40], the odometry key frames obtained by utilizing onboard visual sensing mechanisms are offloaded to a central server. Moreover, to obtain an optimized solution by discovering overlaps, the bag-of-words approach is utilized.

On the other hand, for swarms to function effectively, especially in conditions where communication range is limited or there are poor communication channels, distributed SLAM is a more appropriate approach. The authors of [12] presented a decentralized visual SLAM approach, utilizing readily accessible datasets, and analyzed how to reduce the transmission data. Further investigation is required in order to achieve significant reduction in data transmission to make the whole system energy-efficient. Another recent approach [41] proposed simplification of data representation and utilizing maximum clique outlier rejection for distributed place recognition and pose graph optimization. The algorithm is able to build 3D metric semantic meshes accurately, and is able to handle loop closure errors that may occur. Ref. [42] utilized the Gauss–Siedel technique, and presented a two-stage distributed approach, where they used object-based models to reduce the communication costs between the robots. Furthering the work in [42], authors in [43] presented DOOR-SLAM, a distributed SLAM technique based on peer-to-peer communication. The presented methodology utilizes a pose graph optimizer to reject false inter-robot loop closures. In [22], the authors employed a lightweight Scan Context descriptor to facilitate swarm SLAM, and presented a two-stage global and locally distributed optimization framework. The presented approach runs on a dataset collected by one robot.

### Collaborative Sensing for Map Building

The approach presented in [44] results in significant improvements in swarm SLAM field while utilizing partial feedback from the leading agents in order to find an efficient path to the destination. However, there is no methodology to detect whether the path to the destination discovered by the agent is the most efficient one. This is due to the fact that in the aforementioned approach, as soon as an agent successfully finds an unobstructed path to the destination goal, it broadcasts the tracker points for the rest of the agents in the swarm to follow to be able to reach the destination. Therefore, the presented methodology of partial swarm SLAM is further enhanced in this work by the constant sharing of findings between agents in the swarm in order to build a map in a collaborative manner.

We now present a collaborative sensing approach whereby individual agents form partial maps of known obstacles discovered by other agents, and utilize this information to find the shortest path to their respective goals. It is to be noted that all agents continue sensing their environment to avoid possible collisions with hitherto unknown obstacles or hidden parts of partially discovered obstacles, continuously passing on any such observation to all other agents to update their world view. Figure 1a shows the general map. Grey objects are unknown obstacles, whereas red objects or red parts of the objects imply detected/partially detected obstacles, respectively. As illustrated in Figure 1b, Robot 1 detects an obstacle while navigating towards its goal. Figure 1c shows a scenario in which Robot 1 partially detected a large obstacle and decides to bypass it, while Robot 2 is following the first waypoint dropped by Robot 1. Upon reaching waypoint 2 or the partially detected obstacle Figure 1d, Robot 2 navigates from the other side of the obstacle to complete the detection. This approach also facilitates finding an alternate route that may be superior to the previously discovered path. Upon detection of the optimal path, the rest of the robots will utilize the available information to locally evaluate the options and select the route that is optimal for themselves, as illustrated in Figure 1e.

## 3. Proposed Approach

Algorithm 1 provides the general pseudocode for navigation and obstacle detection. All agents execute this top-level algorithm locally by utilizing their onboard processing units. In the beginning of the mission, all the agents are assigned IDs and connection between them is set up in a leader–follower manner. A global leader is declared, and in a hierarchical manner, the respective leaders are connected to the immediate respective followers, in the initialization phase of the algorithm. Afterwards, the respective agent starts navigation if it has not reached the designated goal, i.e., |$\Gamma_{S}^{i}-\Gamma_{G}$| > ρ, where $\Gamma_{S}^{i}$ and $\Gamma_{G}$ are the coordinates of the *i*th agent and the goal, respectively, and ρ is the radius defined around the goal point to handle any errors that may occur while acquiring the coordinates. If the agent has not yet reached the desired goal, and any obstacle(s) is(are) detected by the onboard ranging sensors, the attributes of the detected obstacle are then stored in an obstacle list (${§}_{\left[i][j:0\to n\right]}$), where *i* stands for the *i*th agent and j:0→n stands for the *j*th obstacle in the list. The respective agent then checks whether this particular object has already been detected by any of the agents that may have navigated through the same path by calling the Merge Objects() function. This Merge Objects() function will perform the following actions if the detected obstacle is present in the obstacle list or if the detected obstacle is not present in the obstacle list. If  the latter is true, then based on the attributes of the detected obstacles in the obstacle list and the newly detected obstacle, it is determined whether the newly detected obstacle is a completely different obstacle or part of a previously partially detected obstacle. These attributes are then passed onto the Shortest Path Obstacle Avoidance() function in order for the agent to be able to perform collision avoidance actively.
**Algorithm 1** Navigation
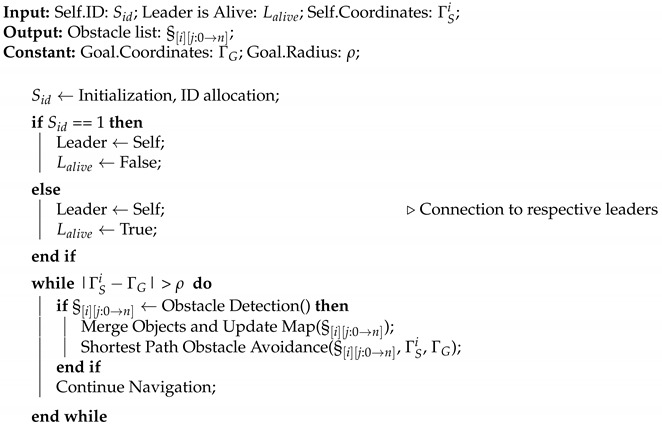


### 3.1. Collision Avoidance and Map Building

When an agent is launched, it executes the obstacle avoidance algorithm to find the shortest path to its destination while avoiding known obstacles. It selects the nearest inflection point as its temporary goal or waypoint, and moves towards this waypoint while continuously sensing its environment for hitherto unseen obstacles. In the case that an obstacle is sensed on its way to the waypoint, the agent performs collision avoidance while simultaneously broadcasting the coordinates of the newly discovered parts of the obstacle. All agents update their individual maps by adding the newly gathered information and run the Merge Objects algorithm to merge multiple sections of the same obstacle into one obstacle. After the agent has moved clear of the obstacle, the process of selecting the waypoint towards its goal is repeated until it completes its mission.

### 3.2. Merge Objects and Update Map

Algorithm 2 starts by checking whether the detected obstacle exists in the obstacle list (${§}_{\left[i\right]}$). Then, for each existing obstacle in the list, the current detected obstacle’s distance is compared with a defined margin of error ($\lambda_{e}$). If the distance or gap between the existing obstacle in the list and the current detected obstacle is equal to or less than $\lambda_{e}$, both obstacles are merged together and considered to be a single obstacle. Otherwise, the current detected obstacle is added to the obstacle list. The updated list is then broadcast by the agent to rest of the agents.
**Algorithm 2** Merge Objects and Update Map
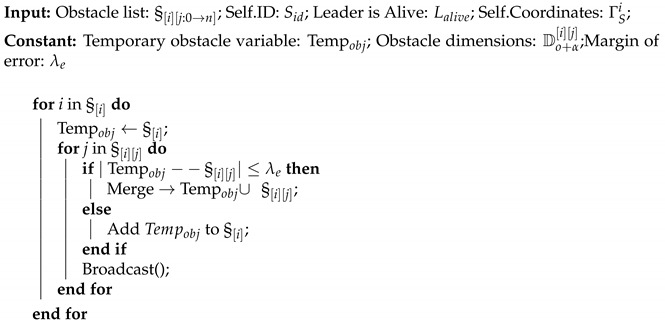


### 3.3. Shortest Path with Obstacle Avoidance

**Figure 2:**
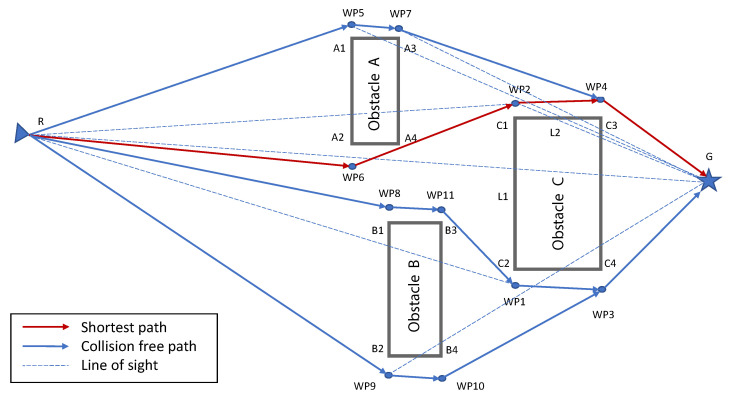
Shortest path planning with obstacle avoidance.

Algorithm 3 shows the pseudocode of the shortest path calculation, as illustrated in Figure 2, while utilizing the collision avoidance algorithm. The algorithm starts by analyzing the attributes of the detected obstacle(s) from the obstacle list, i.e, ${§}_{\left[j:0\to n\right]}$ (Line 1), where $D_{o}^{j}$ is the j th obstacle’s distance from the agent, ${∠}_{o}^{j}$ is the angle at which the obstacle lies, and $D_{o+\alpha }^{j}$ represents the obstacle’s dimensions (*o*: 0 →α). The agent in question then updates the Euclidean distance (E) to the goal from its current position, i.e., $\Gamma_{G}$ and $\Gamma_{S}^{i}$, respectively (Line 2). Afterwards, for each detected obstacle, it is checked whether the obstacle poses a potential collision risk, i.e., lies within the planned trajectory of the agent. This is achieved by analyzing whether the obstacle’s detected dimensions intersect E at any point (Lines 3–5). It is important to note here that the agent may or may not be able to detect the complete obstacle, as the obstacle may be larger than the detection range of the onboard sensor system of the agent. In either case, the extreme edges of the obstacle that are visible to the agent are recorded. If the straight line passes through any of the detected obstacle(s), then based on the available information, the agent calculates new waypoints for collision-free navigation (Line 6). Finally, the waypoint closest to the calculated shortest path is selected as a temporary goal for the agent to navigate towards (Line 12).

**Figure 3:**
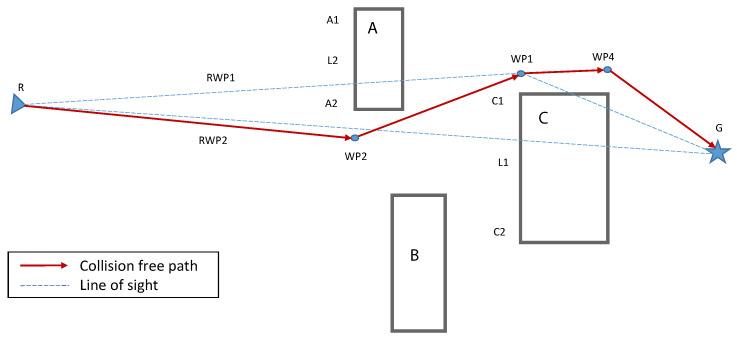
Immediate waypoint selection.

Here, we present two methods of path selection with obstacle avoidance, namely, shortest path planning with obstacle avoidance (SP-OA) and immediate waypoint selection with obstacle avoidance (IWp-OA). First, we explain shortest path planning with the obstacle avoidance algorithm with reference to Figure 3. Consider that a robot at position R is planning its path towards its goal G. Furthermore, its map of the world is populated by three obstacles, A, B, and C, that have been discovered by earlier robots. The steps taken by the said robot, as described in the algorithm Y, are as follows:
**Algorithm 3** Shortest path with Obstacle Avoidance
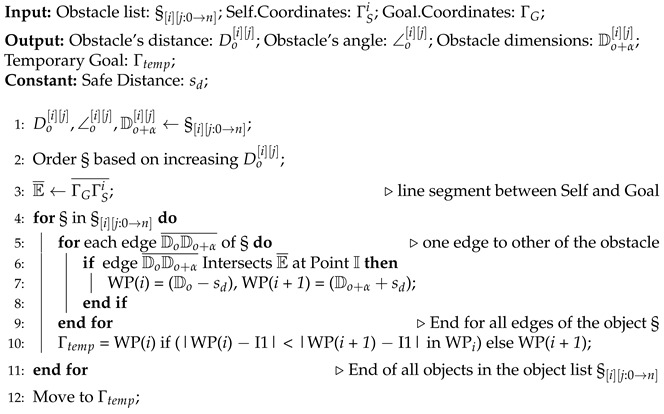

1. The robot draws a straight line from its current position R to its goal G to determine whether it intercepts any edge of the known obstacles. In the instant case, the line $\bar{RG}$ is intercepted by the edge $\bar{C_{1}C_{2}}$ of the obstacle C at point I1.
2. Next, it chooses two waypoints that are a minimum safe distance away from vertices C1 and C2; let us call these W1 and W2. Possible paths are $\bar{RW_{1}}$ followed by $\bar{W_{1}G}$; and $\bar{RW_{}}$ followed by $\bar{W_{2}G}$.
3. The robot iterates steps 1 and 2 above for all possible path segments, always progressing from left to right, creating a new waypoint for each edge that intercepts any path, and repeating steps 1 and 2.
4. We now have a directed acyclic graph (DAG) with multiple obstacle-free paths from the robots current position to the goal.
5. The robot now selects the first waypoint on shortest obstacle-free path as its temporary goal and starts moving towards it.

This algorithm will be re-run every time a new obstacle is discovered, a hidden part of a known obstacle is discovered by the robot’s onboard sensors, or such a finding is communicated by other robots in the swarm. As an alternate approach in the interest of saving computation time and energy, we propose a simplified version of the above algorithm. In this approach, the robot works backwards from the goal, choosing the shorter of two paths at each intersection upon reaching an obstacle. The simplified approach is explained below with reference to Figure 3.

1. The robot draws a straight line from its current position R to its goal G and determines whether it intercepts any edge of the known obstacles. In the instant case, the line $\bar{RG}$ is intercepted by the edge $\bar{C_{1}C_{2}}$ of the obstacle C at point I1.
2. Since the top left corner C1 is nearer to I1 than the bottom left corner C2, it decides to circumvent the obstacle from the top left corner C1. It chooses a waypoint that is a minimum safe distance away from C1, let us call it W1, and sets this waypoint as its temporary goal.
3. It repeats steps 1 and 2 above; this time, the line $\bar{RW_{1}}$ is intercepted by the edge $\bar{A_{1}A_{2}}$ of obstacle A at point I2. The robot avoids obstacle A from bottom left corner A2 and chooses a waypoint WP2 that is a minimum safe distance away from vertex A2. The robot now sets its temporary goal to WP2.
4. The robot repeats step 1 by drawing a straight line $\bar{RW_{2}}$. Since this line is not intercepted by any known obstacle, the algorithm finishes, and the robot starts moving towards its temporary goal WP2.

**Figure 4:**
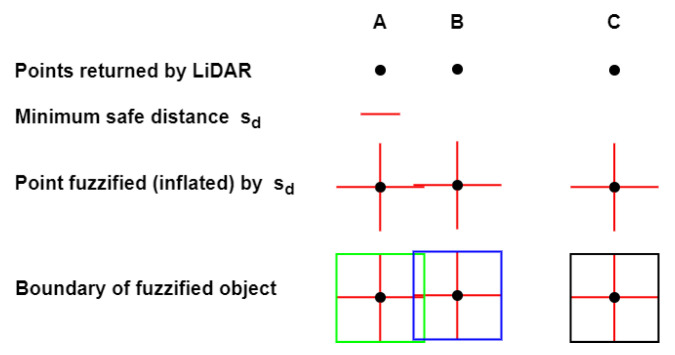
Concept of fuzzification.

The concept of fuzzification of objects is exemplified in Figure 4. In this example, three points, A, B and C, are returned by the LiDAR sensor. The level of inflation or minimum safe distance $s_{d}$ is shown by the red line. The three points are fuzzified in all directions by the level of inflation, shown as red lines in the four dimensions around each point. Next, rectangular objects are created that encompass all edges of the fuzzified points. The objects that overlap, or are adjacent, are merged into one object. Thus, in Figure 4, points A and B are assumed to belong to one object, while point C probably belongs to a second object.

Since there may be other obstacles in the way that are yet to be discovered, planning a complete path to the final goal may be an unnecessary effort at this stage. Additionally, for this reason, the robots continually scan their immediate environment for any hitherto unseen obstacles. They also continue listening to broadcasts from other robots for information about a discovery of a new obstacle or the discovery of further sections of a known obstacle. In either case, the path planning algorithm is executed again to ensure that the path followed by the robot is obstacle-free.

## 4. Simulation Results

A two-dimensional XY plane, i.e., all the objects (robots and obstacles) are at the same altitude, of 12 km × 6 km is used, with unknown obstacles randomly scattered in space. The number of agents are set to ten and are launched from the same coordinates one after the other. Robots move with a maximum speed of 72 km per hour, or 20 m per second. Python graphics are utilized for simulation purposes. For simulation and testing, we utilized the mathematical models of differential drive robots, and further equipped them with the output of a simulated LiDAR sensor in a two-dimensional plane. The following is a kinematic model of a differential drive robot:

(1) $$\begin{array}{c} \begin{array}{c} \dot{x}=vcos\theta ,\\ \dot{y}=vsin\theta ,\\ \dot{\theta }=\frac{v_{\Delta }}{W} \end{array} \end{array}$$

where $\dot{x}$ and $\dot{y}$ are the x and y positions of the robot, *v* is the velocity, and $\dot{\theta }$ is the heading angle of the robot.

The following equation is utilized to calculate the turning curve of the robot:

(2) $$\Delta V=\frac{v_{r}-v_{l}}{W}$$

where ΔV is the difference between the left and the right wheel speed, $v_{r}$ and $v_{l}$ are the right and the left speeds, respectively, and *W* is the width of the robot.

In order to show the progress of the swarm graphically, the field was scaled to fit the available screen resolution, each pixel representing 10 m. The following are the initial conditions and assumptions defined for our work:

1. The robots pass through a vast passage area between the launch zone and delivery zone.
2. There are randomly placed multiple obstacles in the passage area.
3. The communication channel between the robots is considered to be ideal and lossless.
4. Utilizing onboard localization methods, the robots obtain their position vector.
5. The range of LiDAR sensors is 100 m.

**Figure 5:**
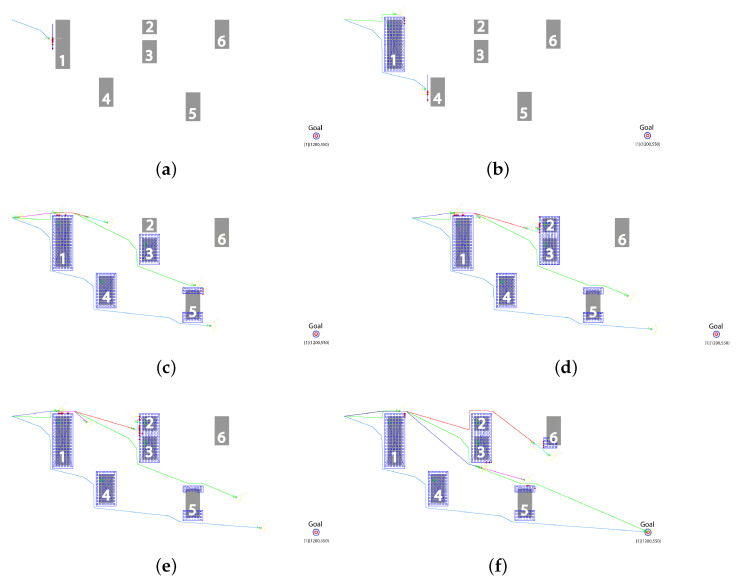
Simulation snapshots: showing the operation of the IWp-OA algorithm. (**a**) Robot 1 avoiding the first detected obstacle from the bottom edge. (**b**) Robot 2 circumvents the obstacle from above to discover an alternate route. (**c**) Robots 1 and 2 have partially discovered obstacle 5. (**d**) Robot 3 discovers obstacle 2, and utilizing fuzzification, combines it with the already discovered obstacle 3. (**e**) Robot 4 chooses the trajectory, by utilizing the broadcast information, to bypass obstacle 3 from below. (**f**) Robot 5 utilizes information provided by all other robots and chooses the optimal trajectory.

Figure 5 shows the effectiveness of utilizing the fuzzy nature of the proposed technique. As can be seen in Figure 5a, Robot #1, on the left of the figure moves along the straight line towards the goal, shown by red circle in the bottom right, and discovers obstacle #1 using a LiDAR sensor; the point cloud is indicated by red dots on the left edge of the obstacle. Using the immediate waypoint with Obstacle Avoidance (IWp-OA) algorithm, it chooses to circumvent the obstacle from below since the the length of edge discovered so far is smaller on this side. As shown in Figure 5b, Robot #2 finds that an already discovered part of obstacle #1 is in the way of the goal, and it decides to circumvent obstacle #1 from above since, by then, most of the left edge is discovered and the top corner seems the shorter way. In another instance, presented in Figure 5c, Robot #1 has discovered, and communicated to other robots, obstacle #4 and the bottom part of obstacle #5. Robot #2 has discovered obstacle #3 and avoids this obstacle by going below it. It has also discovered the top part of obstacle #5. Utilizing the already available information, Robot #3 initially aims to go over the top of obstacle #3, and later discovers obstacle #2; however, since the gap between obstacle #2 and obstacle #3 is narrower than the minimum safe distance, the two are merged and recorded as one obstacle, shown by the blue grid encompassing both obstacles in Figure 5d. As robot #3 is already near the top edge of the merged obstacle, as shown in the simulation screenshot in Figure 5e, it decides to circumvent it from above. Robot #4, after clearing obstacle #1 from above, finds the merged obstacle in its way and decides to bypass it from below. Similarly, Figure 5f shows that Robot #5, or any other following robots, benefits from the information gathered by earlier robots and chooses to pass over the top right corner of obstacle #5, resulting in the shortest path.

**Figure 6:**
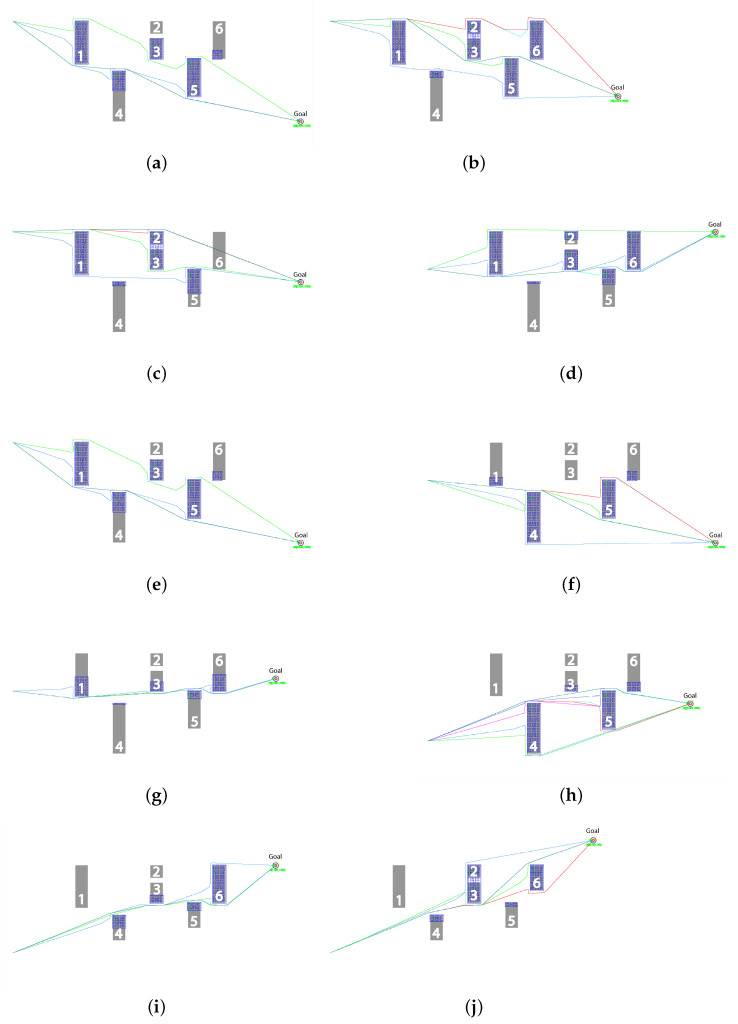
Simulation results: different experimental scenarios. (**a**) Experimental scenario 1. (**b**) Experimental scenario 2. (**c**) Experimental scenario 3. (**d**) Experimental scenario 4. (**e**) Experimental scenario 5. (**f**) Experimental scenario 6. (**g**) Experimental scenario 7. (**h**) Experimental scenario 8. (**i**) Experimental scenario 9. (**j**) Experimental scenario 10.

Similarly, Figure 6 shows the trends of the routes taken by robots from different experimental setups with different launch and goal coordinates. Figure 6a,b show the overall traces of the robots in the swarm from launch to goal, where the launch and goal are slightly towards the center of the initial obstacles. Figure 6c shows the detection and resultant trend when the launch and goal are both moved towards one side of the obstacles. All the robots take similar routes. The robots 1 and 2 performed the optimization for finding an efficient path; afterwards, the rest of the robots followed the discovered optimal path, as shown. Figure 6d shows the trend when the goal is moved diagonally to the other side with same placements of the obstacles, and Figure 6e shows the optimization trend when the obstacles are randomly relocated. Figure 6f,g show the optimization trend over time by the robots when the goal is moved from one point to another while keeping the launch coordinates the same. Similarly, Figure 6h–j show the overall trend while covering all possible scenarios for testing the efficiency of the proposed DCP-SLAM technique.

**Figure 7:**
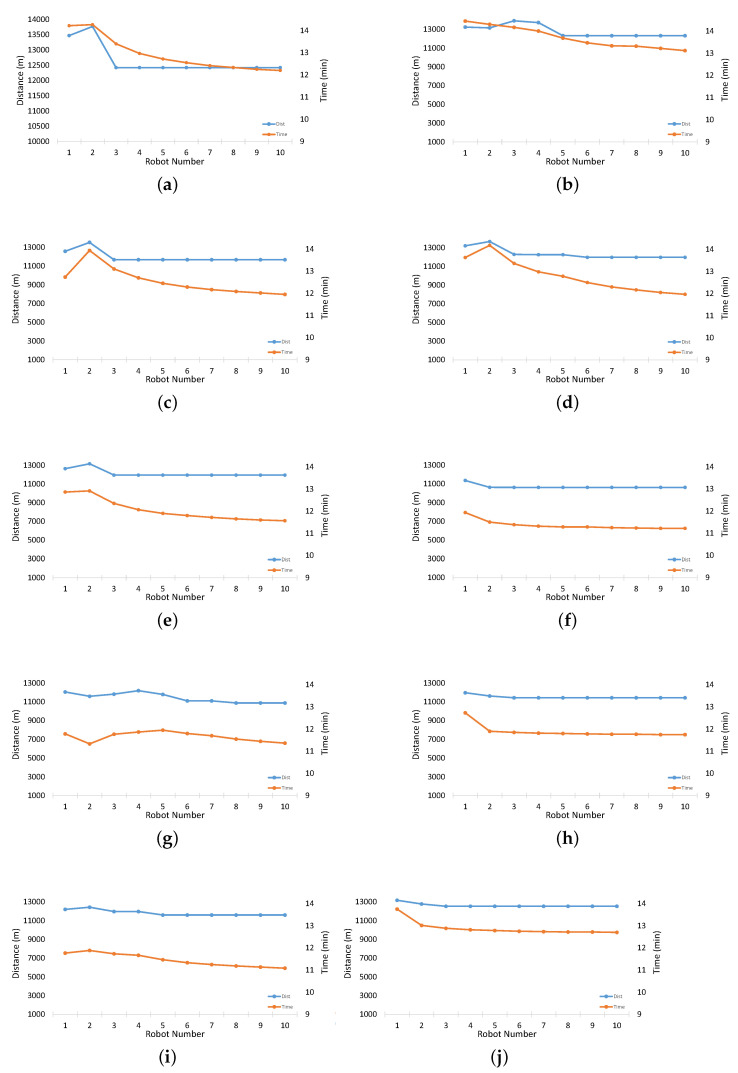
Simulation results: results for respective experiments, showing the total distance traveled by each robot along with the time it took to reach the goal. (**a**) Experimental scenario 1. (**b**) Experimental scenario 2. (**c**) Experimental scenario 3. (**d**) Experimental scenario 4. (**e**) Experimental scenario 5. (**f**) Experimental scenario 6. (**g**) Experimental scenario 7. (**h**) Experimental scenario 8. (**i**) Experimental scenario 9. (**j**) Experimental scenario 10.

Figure 7 shows the distances traveled by individual robots from mission start until they reach the goal of the experiments performed in Figure 6. As reflected, the proposed algorithm manages to find the optimal path to the goal relatively effectively and aggressively.

**Figure 8:**
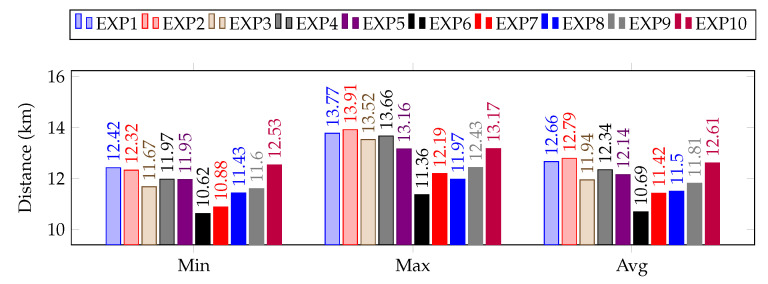
Distance (minimum, maximum, and on average) traveled by the swarm in all experimental scenarios.

**Figure 9:**
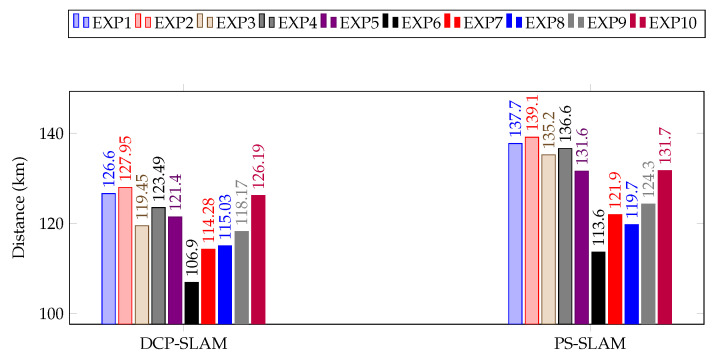
Comparative results of distance traveled by the swarm as a whole.

Figure 8 shows the maximum and minimum distance traveled by a single robot in each experimental scenario, along with the average distance the whole swarm traveled. As is evident from the results, utilizing the proposed approach optimizes the traveling distance relatively quickly as compared to the previously proposed PS-SLAM technique [44] or non-collaborative methods. The results shown in Figure 9 clearly indicate the efficiency in terms of distance traveled by individual robots and, consequently, the swarm as a whole, of the proposed DCP-SLAM technique as compared to the PS-SLAM technique. Evidently, significant consumption is reduced while utilizing the proposed approach, as the distance the swarm traveled is reduced on average by around 10 km. This amounts to an efficiency increase by approximately 10% over the previously developed algorithm [44].

Table 1, Table 2 and Table 3 show total communication cost of each robot in number of messages, Table 1, in number of bytes transmitted, Table 2, and energy consumed in transmission, Table 3. We compare two scenarios for all experiments, firstly, where a robot transmits the point cloud every time it detects an obstacle, resulting in high communication cost for each robot and secondly, where a robot transmits its list of vertices of all obstacles detected so far only when it discovers a new object or hitherto unseen part of an object. As can be seen from the three tables, the latter scheme results in significant saving in communication cost. Since obstacles are gradually discovered and communicated by leading robots, the robots towards the tail end of the swarm have almost zero communication cost.

## 5. Conclusions and Future Work

In this article, we present a methodology for finding an optimal solution for the navigation of a swarm of autonomous robots in environments with no prior map information, utilizing only local onboard sensors for observational purposes and inter-robot communication for the sharing of observations in a concise manner. In the presented technique, robots utilize their onboard ranging sensors to detect and avoid close-range obstacles while navigating towards their goal. The approach works by utilizing the information regarding maps built by leading robots for optimizing the routes for rest of the robots in the swarm. Interestingly, this partial map building approach is robust against communication failure of one or more robots, since the followers build upon whatever information is at hand and utilize onboard sensors to augment it. Thus, any gaps owing to lost information are quickly filled by followers. We have simulated various situations with multiple objects obstructing the straight path to the destination, and show that the proposed approach results in the swarm learning its environment in sufficient detail with the passage of few leading robots so that the remaining members of the swarm can find an optimal path.

Thus, considering the arrival of the whole swarm at the destination as the mission to be carried out with optimal path traversal, some of the leading robots end up taking longer routes and discovering hidden parts of partially discovered obstacles. This additional information is shared with the rest of the swarm, and results in a more informed choice of route by following robots. This results in optimal path selection for the followers and up to 13% saving in the travel path for individual robots. Furthermore, utilizing the proposed approach results in efficiency of up to 11% in traveling distance for the swarm as a whole.

Another contribution of this work is that communication cost is limited to transmitting only significant new discoveries rather than sending the whole point cloud with each detection event of LiDAR by each member of the swarm. This is a direct result of the fuzzification and merging of detected obstacles that reduces the number of obstacles. As long as an obstacle is within the sensing range of a robot’s LiDAR sensor, it receives several detection points in the point clouds for each scan. If this detection data were to be communicated to the rest of the swarm, it would result in high communication cost. Instead, the robot performs several preprocessing steps before transmission. Firstly, it evaluates whether a detection point belongs to an already detected object or not. Here, our fuzzification of the detected objects helps in returning affirmative answers for very close points. If the answer of the first step is negative, it is assumed that the said point belongs to an undetected object. The aforementioned point is fuzzified to form an object with dimensions equal to the minimum clearance distance, and the resulting object is added to the obstacle list. Next, the whole obstacle list is scanned to determine whether any two objects are adjacent or overlapping, merging such objects into one larger object. Since the obstacle list is maintained in a sorted order, only one scan is sufficient to merge all adjacent or overlapping objects. After this final step of merging, the updated obstacle list is broadcast to the rest of swarm. This scheme results in the saving of communication cost and economy of associated communication energy.

In our future work, we aim to further develop the proposed methodology by extending the approach to dynamic environments with moving obstacles. Furthermore, other interesting aspects to analyze will be the effects of communication delays, IMU drifts, and other environmental disturbances in dynamic environments.

## Figures and Tables

**Table 1:** Number of message transmissions.

Exp	1		2		3		4		5		6		7		8		9		10	
**Robot**	**Raw**	**Obj**	**Raw**	**Obj**	**Raw**	**Obj**	**Raw**	**Obj**	**Raw**	**Obj**	**Raw**	**Obj**	**Raw**	**Obj**	**Raw**	**Obj**	**Raw**	**Obj**	**Raw**	**Obj**
1	29,340	105	32,353	154	29,907	245	29,187	185	22,097	101	30,606	167	20,900	140	26,582	160	16,420	131	27,225	140
2	27,393	89	30,766	105	35,216	80	35,197	107	20,523	35	21,868	42	15,338	0	19,078	11	17,867	32	18,770	10
3	12,182	0	23,914	38	18,781	0	15,495	0	11,987	0	22,112	1	26,824	82	18,704	1	14,270	56	18,717	1
4	12,182	0	22,402	0	18,781	0	15,854	5	11,987	0	22,112	0	22,022	10	18,700	0	14,271	0	18,717	0
5	12,182	0	19,030	0	18,781	0	15,854	0	11,987	0	22,112	0	23,951	1	18,700	0	10,052	0	18,717	0
6	12,182	0	19,030	0	18,781	0	11,092	0	11,987	0	22,112	0	16,933	56	18,700	0	10,052	0	18,717	0
7	12,182	0	19,030	0	18,781	0	11,092	0	11,987	0	22,112	0	16,933	0	18,700	0	10,052	0	18,717	0
8	12,182	0	19,030	0	18,781	0	11,092	0	11,987	0	22,112	0	15,726	2	18,700	0	10,052	0	18,717	0
9	12,182	0	19,030	0	18,781	0	11,092	0	11,987	0	22,112	0	15,726	0	18,700	0	10,052	0	18,717	0
10	12,182	0	19,030	0	18,781	0	11,092	0	11,987	0	22,112	0	15,726	0	18,700	0	10,052	0	18,717	0

**Table 2:** Number of kB (kilobytes) transmitted.

Exp	1		2		3		4		5		6		7		8		9		10	
**Robot**	**Raw**	**Obj**	**Raw**	**Obj**	**Raw**	**Obj**	**Raw**	**Obj**	**Raw**	**Obj**	**Raw**	**Obj**	**Raw**	**Obj**	**Raw**	**Obj**	**Raw**	**Obj**	**Raw**	**Obj**
1	82.15	5.46	90.59	8.01	83.74	12.74	81.72	9.62	61.87	5.25	85.69	8.68	58.52	7.28	74.43	8.32	45.98	6.81	76.23	7.28
2	76.70	4.63	86.15	5.46	98.61	4.16	98.55	5.56	57.46	1.82	61.23	2.18	42.95	0	53.42	0.57	50.03	1.66	52.56	0.52
3	34.11	0	66.96	1.98	52.59	0	43.39	0	33.56	0	61.91	0.05	75.11	4.26	52.37	0.05	39.96	2.91	52.41	0.05
4	34.11	0	62.73	0	52.59	0	44.39	0.26	33.56	0	61.91	0	61.66	0.52	52.36	0	39.96	0	52.41	0
5	34.11	0	53.28	0	52.59	0	44.39	0	33.56	0	61.91	0	67.06	0.05	52.36	0	28.15	0	52.41	0
6	34.11	0	53.28	0	52.59	0	31.06	0	33.56	0	61.91	0	47.41	2.91	52.36	0	28.15	0	52.41	0
7	34.11	0	53.28	0	52.59	0	31.06	0	33.56	0	61.91	0	47.41	0	52.36	0	28.15	0	52.41	0
8	34.11	0	53.28	0	52.59	0	31.06	0	33.56	0	61.91	0	44.03	0.10	52.36	0	28.15	0	52.41	0
9	34.11	0	53.28	0	52.59	0	31.06	0	33.56	0	61.91	0	44.03	0	52.36	0	28.15	0	52.41	0
10	34.11	0	53.28	0	52.59	0	31.06	0	33.56	0	61.91	0	44.03	0	52.36	0	28.15	0	52.41	0

**Table 3:** Energy consumed in transmission (mJ).

Exp	1		2		3		4		5		6		7		8		9		10	
**Robot**	**Raw**	**Obj**	**Raw**	**Obj**	**Raw**	**Obj**	**Raw**	**Obj**	**Raw**	**Obj**	**Raw**	**Obj**	**Raw**	**Obj**	**Raw**	**Obj**	**Raw**	**Obj**	**Raw**	**Obj**
1	82.15	0.55	90.59	0.80	83.74	1.27	81.72	0.96	61.87	0.53	85.69	0.87	58.52	0.73	74.43	0.83	45.98	0.68	76.23	0.73
2	76.70	0.46	86.14	0.55	98.61	0.42	98.55	0.56	57.46	0.18	61.23	0.22	42.95	0	53.42	0.06	50.03	0.17	52.56	0.05
3	34.11	0	66.96	0.19	52.59	0	43.39	0	33.56	0	61.91	0.01	75.11	0.43	52.37	0.01	39.96	0.29	52.41	0.01
4	34.11	0	62.73	0	52.59	0	44.39	0.03	33.56	0	61.91	0	61.66	0.05	52.36	0	39.96	0	52.41	0
5	34.11	0	53.28	0	52.59	0	44.39	0	33.56	0	61.91	0	67.06	0.01	52.36	0	28.15	0	52.41	0
6	34.11	0	53.28	0	52.59	0	31.06	0	33.56	0	61.91	0	47.41	0.29	52.36	0	28.15	0	52.41	0
7	34.11	0	53.28	0	52.59	0	31.06	0	33.56	0	61.91	0	47.41	0	52.36	0	28.15	0	52.41	0
8	34.11	0	53.28	0	52.59	0	31.06	0	33.56	0	61.91	0	44.03	0.01	52.36	0	28.15	0	52.41	0
9	34.11	0	53.28	0	52.59	0	31.06	0	33.56	0	61.91	0	44.03	0	52.36	0	28.15	0	52.41	0
10	34.11	0	53.28	0	52.59	0	31.06	0	33.56	0	61.91	0	44.03	0	52.36	0	28.15	0	52.41	0

## Data Availability

Not applicable.
